# Identification of circRNA-miRNA-mRNA network in luminal breast cancers by integrated analysis of microarray datasets

**DOI:** 10.3389/fmolb.2023.1162259

**Published:** 2023-04-28

**Authors:** Yixiang Huang, Mingping Qian, Juhang Chu, Lei Chen, Wei Jian, Gang Wang

**Affiliations:** Department of Thyroid and Breast Surgery, Shanghai Tenth People’s Hospital, Tongji University School of Medicine, Shanghai, China

**Keywords:** network, luminal breast cancer, circRNA, tamoxifen resistance, hsa_circ_0086735-miR-1296-5p-STAT1

## Abstract

**Introduction:** Circular RNAs (circRNAs) regulatory network is important in human cancer. We, therefore, mapped the regulatory networks driven by circRNA in luminal-subtype breast cancer.

**Methods:** Breast cancer-related microarray datasets from GEO database were analyzed for the differentially expressed circRNAs, miRNAs, and mRNAs. The potential downstream RNAs were collected using Circular RNA Interactome or Targetscan database. Protein-protein interaction (PPI) analysis was performed for the filtered genes to identify hub genes. The functions were annotated by the Gene Ontology (GO) and Kyoto Encyclopedia of Genes and Genomes (KEGG) enrichment analysis. CircRNA-miRNA-mRNA networks were mapped using Cytoscape software. Hsa_circ_0086735-miR-1296-5p-STAT1 axis was used for verification. The expression levels of hsa_circ_0086735, miR-1296-5p, and STAT1 mRNA were confirmed by qRT-PCR in luminal-subtype tissues and cell lines. The interactions among them were verified by Luciferase reporter assay and RNA pull-down assay. Cell proliferation and apoptosis were assayed. Overall and distant metastasis-free survival was analyzed.

**Results:** A total of 70 genes were finally targeted and enriched in multi-process and multi-pathway. Networks containing 96 circRNA-miRNA-mRNA axes were constructed. Hsa_circ_0086735 and STAT1 mRNA was upregulated in luminal breast cancer, while miR-1296-5p was downregulated. Hsa_circ_0086735-miR-1296-5p-STAT1 axis promotes breast cancer progression and contributes to tamoxifen resistance. High hsa_circ_0086735 was associated with poor overall and distant metastasis-free survival.

**Discussion: **This study identified the hsa_circ_0086735-miR-1296-5p-STAT1 as an important regulatory axis in luminal-subtype breast cancer, aiding to determine potential therapeutic targets.

## Introduction

Breast cancer is a global female malignancy and the leading cause of female cancer deaths (Loibl et al., 2021). It is a molecularly heterogeneous disease, identified as at least five molecularly distinct subtypes based on gene expression (Tsang and Tse, 2020). These molecular subtypes have distinct prognostic values across multiple treatment settings (Cheang et al., 2009). Among these subtypes, luminal breast cancers are termed hormone-receptor-positive breast cancer, including estrogen receptor (ER)-positive-luminal A (luminal A) and ER-positive-luminal B (luminal B) (Pellegrino et al., 2021). Luminal breast cancer is the most common type in women diagnosed with breast cancer, characterized by a high risk of relapse (Adachi et al., 2016). Currently, ER status is one predictive marker with an associated targeted therapy. The therapy choice for two-thirds of luminal breast cancer is endocrine therapy with tamoxifen or aromatase inhibitors (Katsura et al., 2022). Endocrine therapy has contributed to a recent decrease in the risk of the composite outcome of recurrence or death in breast cancer (Kerr et al., 2022). However, the emergence of acquired tamoxifen resistance, in approximately 40% of patients, is the major problem (Mishra et al., 2021). Although endocrine therapy would benefit many lower-risk patients, it may fail to prevent recurrence in some patients with high-risk resistance. Endocrine resistance can emerge from impaired estrogen signaling, MYC overexpression, amplification of cyclin D1 or MDM2 gene, lower activity of CYP2D6, or aberrant promoter methylation that activates Akt/mTOR pathway or other estrogen-independent growth-promoting pathways (Treeck et al., 2023). Thus, there is a need to unveil the underly mechanism of subtype breast cancer, the novel therapy targets overcoming the tamoxifen resistance, and the biomarkers to circumvent poor disease prognosis.

Though the traditional notion of gene regulation is centered around DNA and mRNAs, the protein-coding region only accounts for 2% of the human whole-genome transcriptome (Kong et al., 2022). A large proportion of the sequence, more than 90%, is found transcription for non-coding RNAs (ncRNAs) (Cohen and Jia, 2014). Though the ncRNAs do not control protein synthesis, they demonstrate their importance in tumorigenesis and tumor progression (Yan and Bu, 2021). micro-RNAs (miRNAs), a set of small ncRNAs of −20–23 nucleotides in length, is a type of well-studied ncRNA now (Ashekyan et al., 2022). Circular RNAs (circRNAs) are a recently understood significant class of ncRNAs, whose 5′ and 3′ ends are covalently attached via “back-splicing” (Yu and Kuo, 2019). CircRNAs demonstrate a more stable potential and are resistant to degradation by exonucleases due to the absence of 5′ or 3′ end (Jeck et al., 2013). Whereas miRNAs silence mRNA and regulate gene expression, circRNAs function through several mechanisms at transcriptional/translational levels (Kristensen et al., 2019). Both miRNAs and circRNAs have various roles in biological process regulation. A pan-cancer analysis has reported circRNA CDR1as acts as a mediator in the alteration of the tumor microenvironment (Zou et al., 2019). Moreover, circRNAs harboring miRNA response elements can sponge miRNAs and interfere with the splicing of other RNAs as endogenous RNAs (ceRNAs) (Kristensen et al., 2019). The binding and inhibition of miRNA by circRNA are one of the most influential and deregulated mechanisms in cancer (Zhang et al., 2021). The possible interaction between circRNAs and miRNAs makes circRNA possible to cross paths with various significant pathways related to cancers, acting as oncogenes or tumor suppressors depending on the function of the miRNA-downstream genes (Ashekyan et al., 2022). As circRNA could sponge to tens of miRNAs and miRNAs have thousands of targeting genes, even a minor dysregulation in the circRNA/miRNA/mRNA pathway could be ruinous (Khan et al., 2021). The circRNA-miRNA-mRNA axis could interfere with many drugs and relate to the progression of many tumors, including breast cancer (Ghazimoradi and Babashah, 2022). Therefore, this impactful axis, including the network resulting from it, needs to be discovered.

In a search for a circRNA-miRNA-mRNA network that might influence luminal subtype and tamoxifen resistance, the related GEO datasets were downloaded and analyzed. The differentially expressed circRNAs, miRNAs, and mRNAs were used to map the network. To elucidate the regulatory and molecular mechanism of the circRNA-miRNA-mRNA axis, cell functional assays, including cell proliferation and cell apoptosis, were performed.

## Materials and methods

### Associated circRNA expression dataset extraction

The GEO database was exhaustively searched for breast-cancer-related microarray datasets. Four GEO datasets, with accession numbers GSE182471, GSE101410, GSE111504, and GSE159980, were selected for the screen of circRNAs dysregulated in tamoxifen-resistant and metastatic luminal breast cancer. Dataset GSE182471 summarizes circRNA expression profiles from five pairs of breast cancer tissue samples and adjacent non-tumor tissue samples by microarray and RT-qPCR. GSE101410 comprises RNA-seq Luminal subtype-specific circRNAs in breast cancer cells. GSE111504 identified the circRNA expression signatures for breast cancer metastasis. GSE159980 is composed of microarray analysis of tamoxifen-sensitive and -resistant cells. Raw expression data were scored and extracted/normalized using the GEO-online tool GEO2R after applying log2 transformation. The overlapped circRNAs among these four datasets were available by VENNY diagram.

### Associated miRNA expression dataset collection

GSE62022, GSE110204, and GSE121172 were analyzed using GEO2R for the luminal-specific miRNAs. The dysregulated miRNAs were subjected to a volcano-diagram presentation. The potential miRNAs for the obtained circRNAs were collected using Circular RNA Interactome (https://circinteractome.nia.nih.gov/index.html). The dysregulated miRNAs from GSE datasets were integrated with the circRNA-targeting miRNAs. GSE48390 was normalized and log2 transformed using GEO2R for differentially expressed genes between the high- and low-risk groups. Genes with absolute logFC values more than 2 and adjusted *p*-value less than 0.05 were screened as significantly differentially expressed.

### Construction of protein-protein interaction networks

Significantly differentially expressed genes were subjected to STRING for the prediction of protein interactions. Networks were constructed using Cytoscape software and the hub genes in the network were identified using cyto-Hubba, a plug-in Cytoscape.

### Functional and pathway enrichment analysis

Gene symbols were transformed for the analysis of gene-centric functional and canonical pathway enrichment analysis using OmicShare Tools.

### Construction of the circRNA-miRNA-mRNA network

Combining RNA interactions and expression levels, the circRNA-miRNA-mRNA networks were established as two distinct sets using Cytoscape software. The circRNA-miRNA-mRNA axes containing hub genes were used for the visualization of final biological networks.

### Patients and sample study

To be eligible for this study, women had to be at their first diagnosis of luminal-subtype breast cancer (Engstrøm et al., 2013). The candidates received no cancer-related therapy before surgery and immediately underwent breast surgery, followed by tamoxifen treatments. The tumor core biopsies, along with the adjacent normal tissues, were obtained from all patients and preserved in RNAlater for circRNA, miRNA, and mRNA expression evaluation. All women included in this study provided written informed consent. The study was approved by the Research Ethics Committee at Shanghai Tenth People’s Hospital, Tongji University School of Medicine. Information on survival time and distant metastasis was obtained from electronic or paper medical records in our institution.

### Cell culture

The cell lines used in the analysis include MCF7, ZR-75-1, MCF7 Tam1, and ZR-75-1 Tam1, purchased from American Type Culture Collection (Rockville, MD, United States). Whereas ZR-75-1 and MCF7 were respectively cultured in 10% FBS-supplemented RPMI-1640 and EMEM, culture media for MCF7 was additionally contained and 10 μg/mL human insulin (Sigma. St. Louis, MO, United States). The tamoxifen-resistant cell lines, MCF-7 Tam1 and ZR-75-1 Tam1, used the same culture media as the parental cell lines, additionally containing 1 µM 4-hydroxytamoxifen (Sigma. St. Louis, MO, United States).

### Transient transfections

Hsa_circ_0086735 siRNA (si-circ), STAT1 siRNA (si-STAT1), and non-silencing control plasmids (si-NC and ctr-STAT1) were purchased from Guangzhou RiboBio. Mi-1296-5p inhibitor (in-miR) and non-silencing control (in-NC) were purchased from Guangzhou Genecopoeia. The above vectors were transfected into cells by transient transfection using Lipofectamine 3,000 transfection reagents from Invitrogen.

### RNA isolation and quantitative real-time PCR (qRT-PCR)

First, total RNA was extracted from tissue homogenate and cells using TRIzol (Life Technologies, Carlsbad, CA, United States). Then, total RNA was purified using the miRNeasy Mini kit (QIAGEN, Hilden, Germany) and assessed for purity using a NanoVue spectrophotometer (Ge BioSciences, United States). Total RNA, combined with anchored oligo (dT)18 primer and reverse transcriptase from the Transcriptor High FidelitycDNA Synthesis Kit (Roche, Germany), was subjected to the first strand synthesis for cDNA. The cDNA samples were examined by qRT-PCR using FastStart™ universal SYBR^®^ Green (Roche) for determination of miR-1296-5p and STAT1 mRNA, using U6 and beta-actin as the endogenous control. For hsa_circ_0086735, total RNA was prepared to the linear RNA using RNase R digestion (Epicentre Technologies, Madison, WI, United States). RNase R-treated RNA was reversely transcribed to cDNA using SuperScript III First Strand Synthesis System (Invitrogen). qRT-PCR was performed using Power Up Sybr Green Master Mix (Thermo Fisher Scientific). Each sample was assayed in triplicate determinations at least.

### Luciferase reporter assay

Luciferase reporter plasmids, including WT-circ, MUT-circ, WT-STAT1, and MUT-STAT1, were constructed by LandM Biotechnology (Guangzhou, China). These reporter plasmids were singly transfected into MCF7 and ZR-75-1 cells with miR-1296-5p mimic or inhibitor. After incubation, each cells lysate was prepared using two kinds of buffers, Firefly luciferase substrate and Firefly luciferase inhibitor/Renilla luciferase substrate, corresponding to the first measure of the Firefly luciferase activity and second measure of the Renilla luciferase activity. Finally, the normalized luciferase activity was calculated by Firefly luciferase activity/Renilla luciferase activity. Data were collected from at least three independent experiments for each subgroup.

### RNA pull-down assay

The combination of miR-1296-5p and hsa_circ_0086735, as well as miR-1296-5p and STAT1, was determined by RNA pull-down assay. The miR-1296-5p probe (biotin-miR) and its negative control (biotin-NC) were constructed by Ribobio. Harvested MCF7 and ZR-75-1 were lysed using lysis buffer and then incubated with a biotin-labeled miT-1296-5p probe and streptavidin agarose magnetic beads. Hsa_circ_0086735 and STAT1 mRNA in miR-1296-5p probe pull-down complex were detected by qRT-PCR. Data were collected from at least three independent experiments for each subgroup.

### Proliferation assays

An appropriate number of cells (1 × 10^5^ of MCF7 and 8 × 10^4^ of ZR-75-1 or ZR-75-1 Tam1) were seeded into a 96-well plate. Different periods after plating, 0, 12, 24, 48, and 72 h for MCF7 and ZR-75-1 and 0, 2, 4, 6, 9 days for ZR-75-1 Tam1, 10 μL of CCK-8 solution (Millipore-Sigma) was added to each well, followed by a 2-h incubation. The optical density of each well was measured at 450 nm using a BioTek spectrophotometer.

For tamoxifen-sensitive studies with the ZR-75-1 tamoxifen-resistant cell line, cells were seeded with increasing tamoxifen concentrations (0–120 μg/mL). After 6 days, cell viability was evaluated by CCK-8 solution (Millipore-Sigma). Data were collected in biological duplicates run in triplicate for each time point.

### Annexin V-FITC/PI double staining for apoptosis

To quantify apoptosis, transfected cells cultured for 48 h were harvested, digested, and pipetted to achieve a uniform single-cell suspension. Then cells were doubly stained with the Annexin V-FITC Apoptosis Detection Kit (Sigma-Aldrich). To score Annexin V and/or PI-positive cells, cells were subjected to analysis on a BD Accuri C6 Plus Flow Cytometer (Franklin Lakes, NJ, United States). All data were obtained from at least three independent culture experiments.

### Statistical analysis

Hsa_circ_0086735, miR-1296-5p, and STAT1 mRNA expression levels were calculated from qRT-PCR CT values using 2^−ΔΔCT^. The paired *t*-test was conducted for comparison of the expression differences between the tumor and normal tissues. Based on the mean value of hsa_circ_0086735 in the enrolled patients’ tissues, patients were classified into a low-expression group and a high-expression group. Chi-square test or Spearman coefficient of correlation was used to compare tumor characteristics across categories of hsa_circ_0086735 expression levels. The prognostic significance of relevant variables was assessed by Cox’s proportional-hazards regression from multivariate analysis. Overall and distant metastasis-free survival was assessed by Kaplan-Meier analysis with the log-rank test. Whereas normally distributed variables were compared using two-sided t-tests, non-normally distributed variables were compared using Mann-Whitney U tests. A *p*-value less than 0.05 was the cutpoint to be considered significant.

## Results

### Differential expression of circRNAs in tamoxifen-resistant luminal breast cancer

**FIGURE 1:**
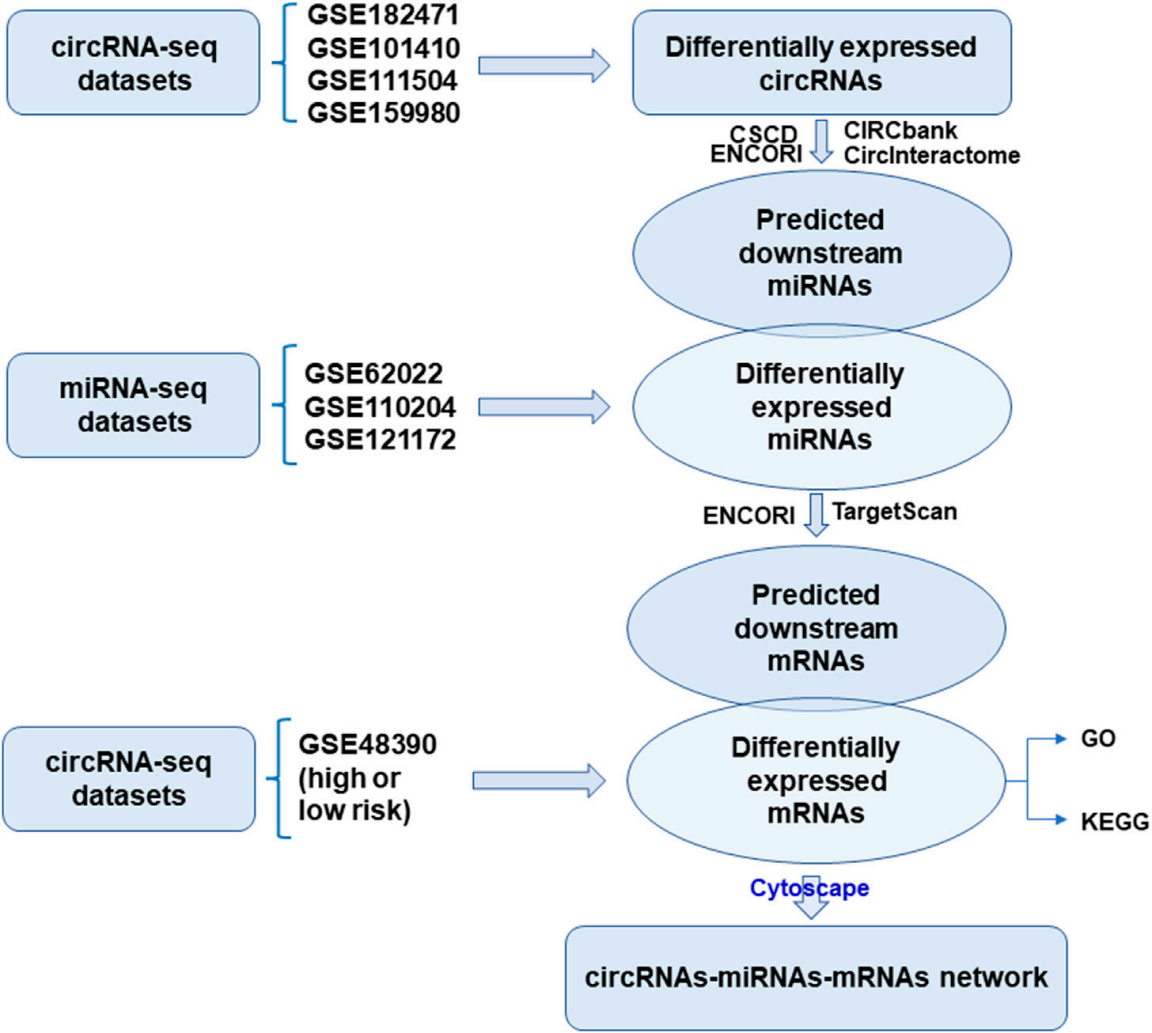
Flowchart of the construction of circRNA-miRNA-mRNA network.

**FIGURE 2:**
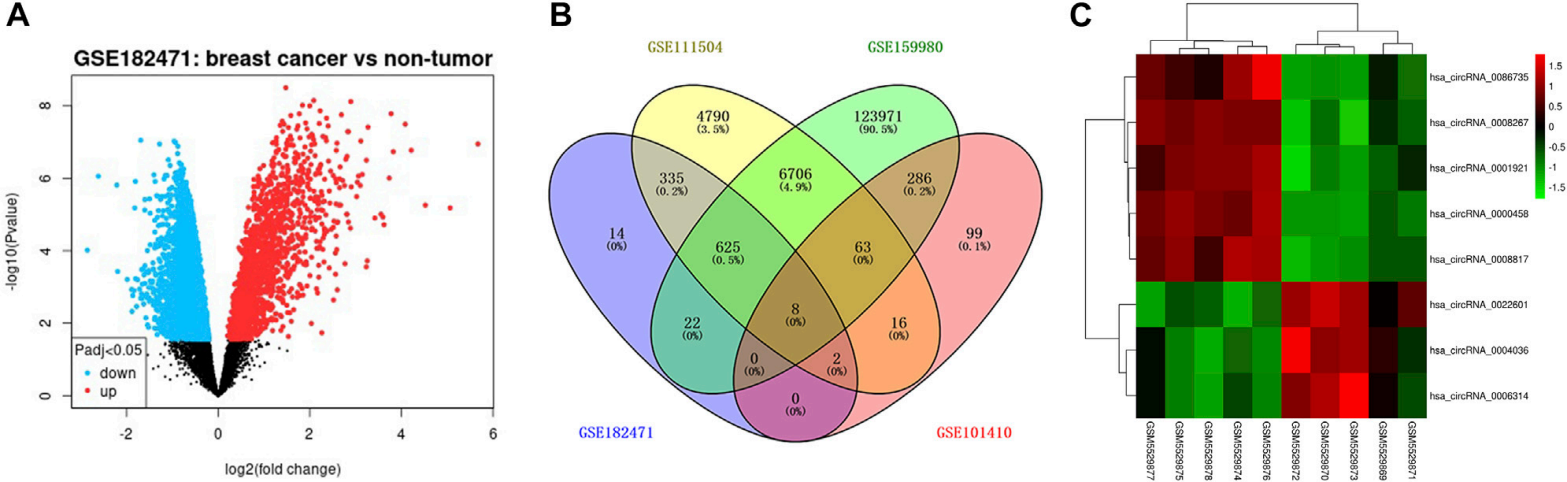
The differentially expressed circRNAs in tamoxifen-resistant and luminal-subtype breast cancer. **(A)** Volcano map of up/downregulated circRNAs in GSE182471 analyzed by GEOR2. The red dots indicated upregulated circRNAs and the blue dots indicated downregulated circRNAs. **(B)** Venn diagrams of the shared differentially expressed circRNAs among GSE182471, GSE111504, GSE159980, and 101,410. **(C)** Heatmap of eight up/downregulated circRNAs according to the expression in GSE182471.

The flowchart of construction for the circRNA-miRNA-mRNA network was shown in Figure 1. To filter differentially expressed circRNAs, GSE18247 was analyzed by GEO2R (Figure 2A). To determine differentially expressed circRNAs in tamoxifen-resistant luminal breast cancer, the circRNAs obtained from GSE182471 were crossed and overlapped with those from GSE101410, GSE111504, and GSE159980. There were eight circRNAs significantly dysregulated in tamoxifen-resistant luminal breast cancer (Figure 2B). The differential expression of these eight circRNAs, hsa_circ_0086735, hsa_circ_0008267, hsa_circ_0001921, hsa_circ_0000458, hsa_circ_0008817, hsa_circ_0022601, hsa_circ_0004036, and hsa_circ_0006314, was presented as a heat map in Figure 2C.

### Differentially expressed miRNAs targeted by the dysregulated circRNAs

**FIGURE 3:**
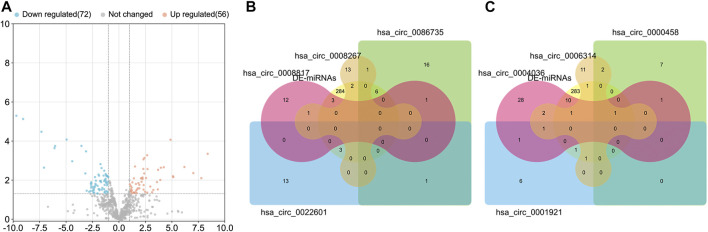
The differentially expressed miRNAs (DE-miRNAs) in luminal-subtype breast cancer. **(A)** Volcano map of the shared miRNAs from GSE32022, GSE110204, and GSE121172. The orange dots indicated upregulated micRNAs and the blue dots indicated downregulated miRNAs. **(B)** and **(C)** Venn diagrams of the overlapped miRNAs among DE-miRNAs and the circRNA-targeting miRNAs.

The luminal-related miRNAs were obtained from GSE32022, GSE110204, and GSE121172, and presented as a Volcano diagram (Figure 3A). The downstream miRNAs of the eight dysregulated circRNAs were collected and overlapped with the luminal-subtype-related differentially expressed miRNAs (DE-miRNAs) (Figures 3B,C). In total, 26 miRNAs were classified as present across the panel of eight dysregulated circRNAs. These target miRNAs were used for further differential analysis.

### The prognostic gene corresponding to differential expressed circRNA-miRNA axes

**FIGURE 4:**
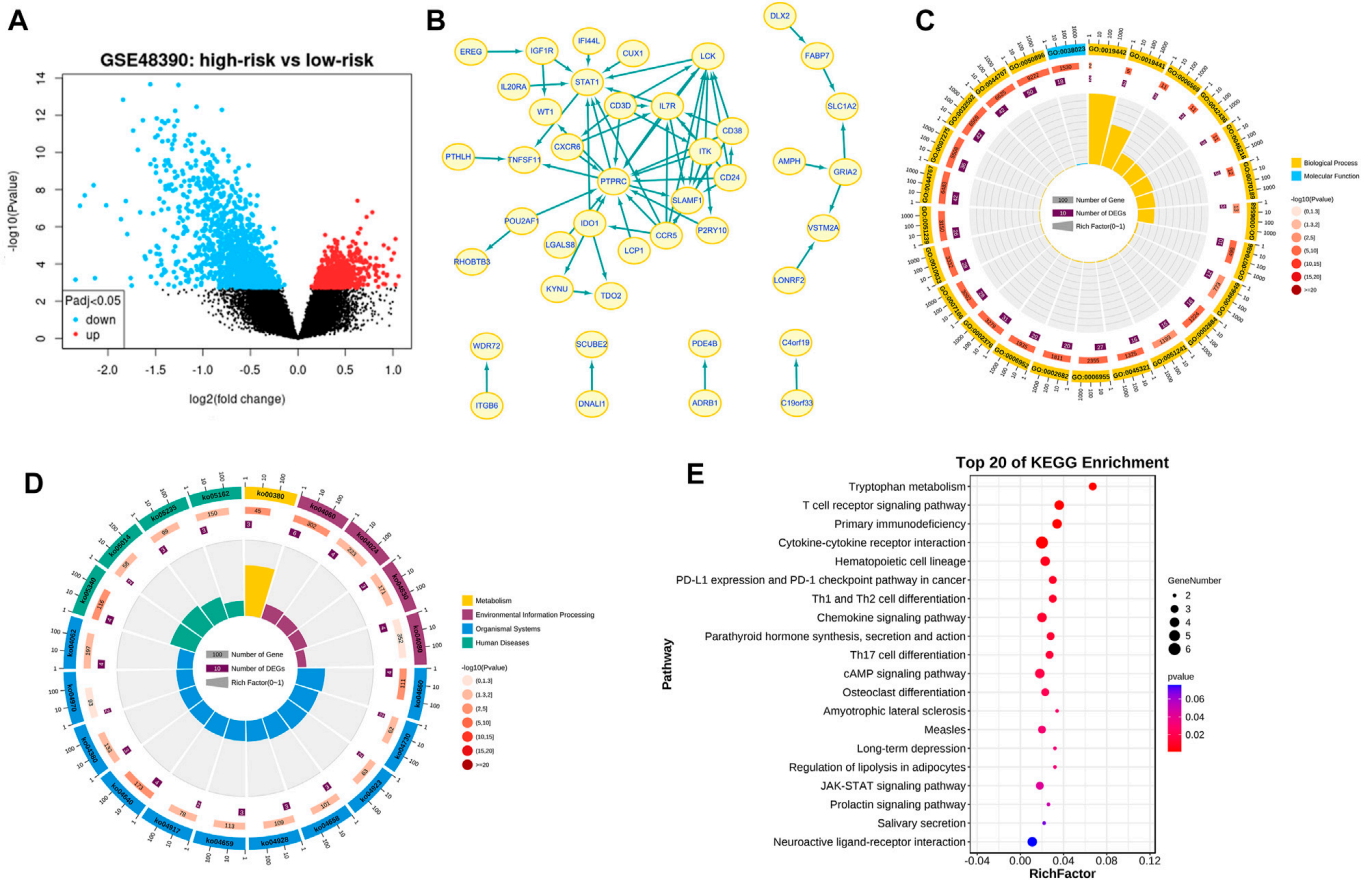
Bioinformatics of the differentially expressed mRNAs. **(A)** Volcano map of differentially expressed mRNAs in GSE48390 analyzed by GEOR2. **(B)** Protein-protein interaction (PPI) networks of the shared mRNA between differentially expressed mRNAs and miRNA-targeting mRNAs. **(C)** The enriched GO terms by the shared mRNAs. **(D)** The enriched KEGG pathways of the shared mRNAs. **(E)** The top 20 pathways enriched by the shared mRNAs.

The prognostic gene signature from GSE48390 for Han Chinese breast cancers was analyzed by GEO2R. A total of 508 differentially expressed and prognosis-related genes were identified (Figure 4A). Subsequently, 70 genes were screened to cover the downstream genes of the above 26 miRNAs, and their PPI was shown in Figure 4B with orphaned nodes deleted. The top 10 hub nodes in the PPI network were identified as PTPRC, IL7R, STAT1, LCK, SLAMF1, CCR5, ITK, CD38, CD3D, and CD24. We further conducted the GO and KEGG pathway enrichment analysis of 70 genes. The GO-enriched results showed that the genes mainly enriched in the main immune system process, developmental process, response to stimulus, and multicellular organismal process (Figure 4C). KEGG enrichment analysis showed that they were enriched for 124 pathways (Figure 4D). In KEGG, the top 20 enriched pathways included PD-L1 expression and PD-1 checkpoint pathway in cancer, cAMP signaling pathway, and JAK-STAT signaling pathway (Figure 4E).

### Maps of the ceRNA (circRNA-miRNA-mRNA) network

**FIGURE 5:**
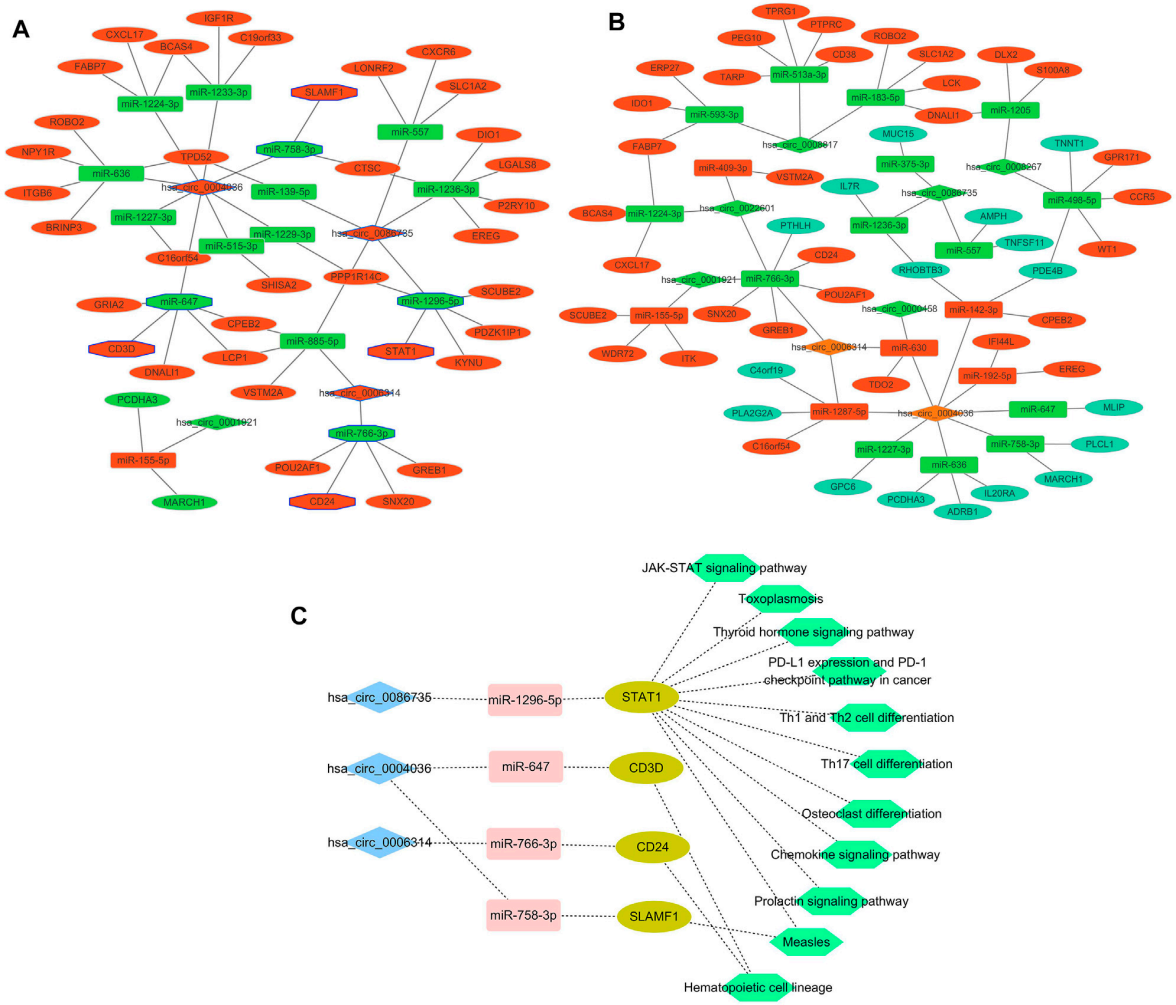
Networks. **(A)** and **(B)** Differentially expressed circRNA-miRNA-mRNA networks. The diamond represented circRNAs, the rectangles miRNAs, and ellipses mRNAs. Red and yellow color represent up- and downregulation in luminal-B tumor tissues, respectively. The black border indicated the pathway derived from the top 10 hub genes. **(C)** Network of the main circRNA-miRNA-mRNA-pathway.

A total of 96 circRNA-miRNA-mRNA axis, including 47 correctly sequenced ones (Figure 5A) and 49 invalid-ordered ones (Figure 5B), were obtained with the above-obtained RNAs. As for the hub genes (Supplementary Table S1), the circRNA-miRNA-mRNA-pathway network was mapped (Figure 5C). Given STAT1 was one of the hub genes that ranked second (Score = 10) in the interaction network by Degree method (Supplementary Table S1), we chose the hsa_circ_0086735-miR-1296-5p-STAT1 axis for further validation.

### Validation of hsa_circ_0086735, miR-1296-5p, and STAT1 expression

**FIGURE 6:**
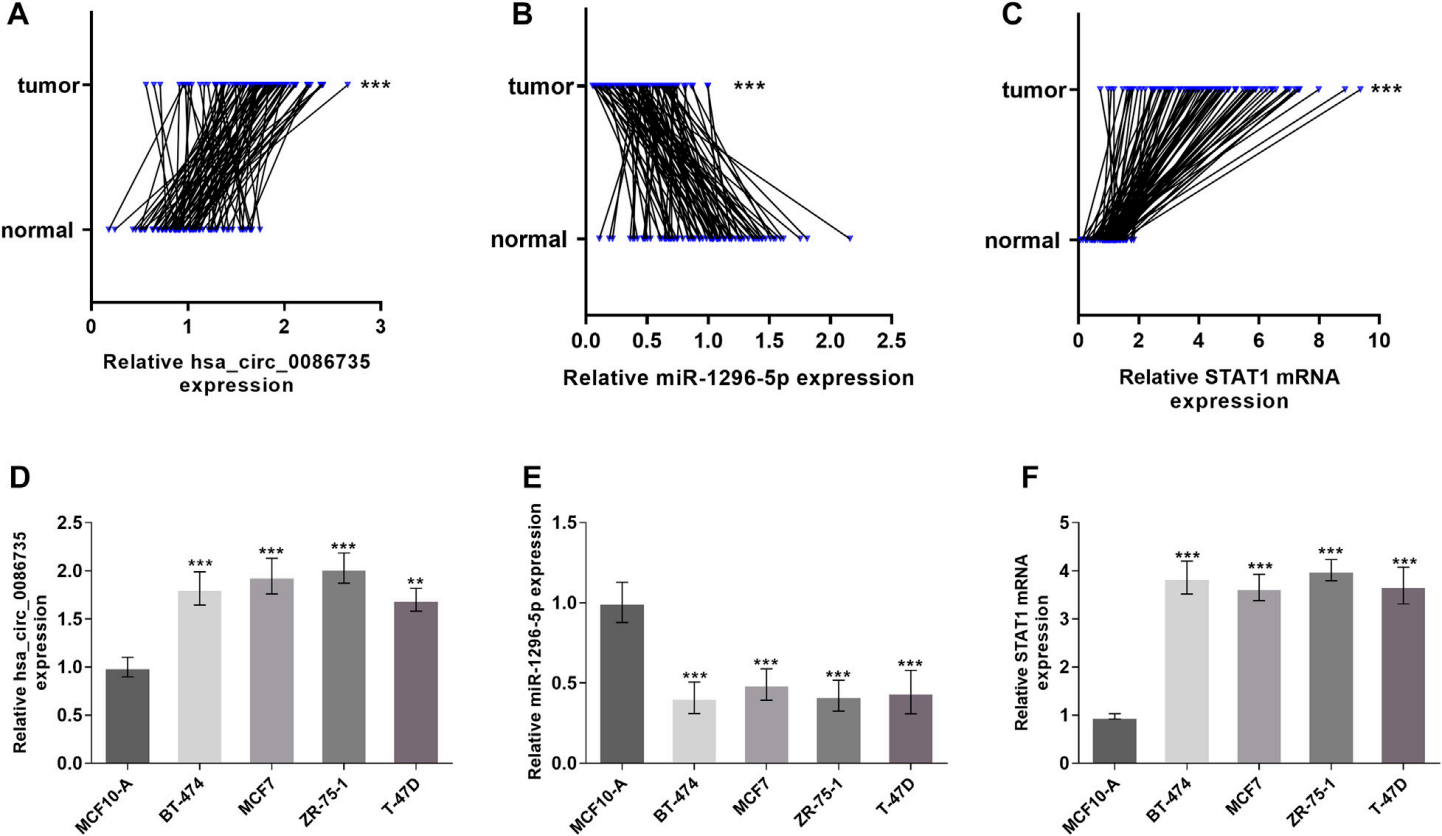
Expression of hsa_circ_0086735 in luminal-subtype breast cancer. Luminal-subtype breast cancer tissues expressed higher hsa_circ_0086735 **(A)**, lower miR-1296-5p **(B)**, and higher STAT1 mRNA **(C)** compared to those in adjacent normal tissues. Expression of hsa_circ_0086735 **(D)**, miR-1296-5p **(E)**, and STAT1 mRNA **(F)** in luminal-subtype cell lines compared to those in MCF10-A. ***p < 0.001.

qRT-PCR was used to validate the expression levels of hsa_circ_0086735, miR-1296-5p, and STAT1 in luminal-subtype breast cancer. Hsa_circ_0086735 was significantly upregulated in luminal breast tumor tissues (*p* < 0.001, Figure 6A), which corresponded to the array data from GSE182471. In contrast, miR-1296-5p was significantly downregulated in the tumor tissues (*p* < 0.001, Figure 6B). STAT1 mRNA was an upregulated mRNA in luminal cancerous tissues (*p* < 0.001, Figure 6C). Luminal-subtype cell lines showed the same altered tends correspondingly (*p* < 0. 01, Figures 6D–F).

### Validation of hsa_circ_0086735-miR-1296-5p-STAT1 axis

**FIGURE 7:**
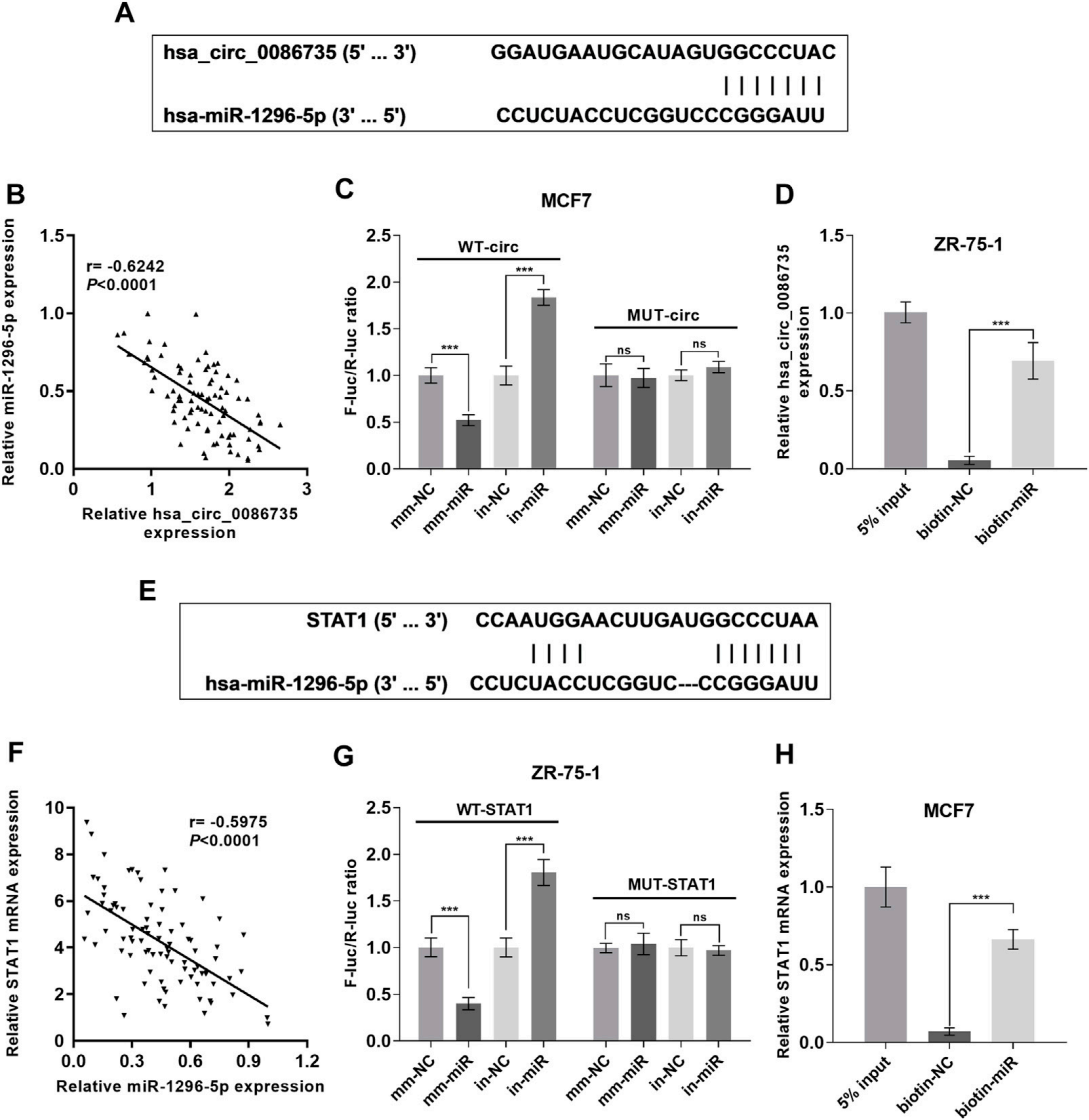
Hsa_circ_0086735 sponged to miR-1296-5p, and miR-1296-5p targeted to STAT1. **(A)** Predicted complementary binding sites of hsa_circ_0086735 against miR-1296-5p. **(B)** The expression level of hsa_circ_0086735 was reversely related to that of miR-1296-5p. **(C)** Luciferase reporter assays confirm the binding between hsa_circ_0086735 and miR-1296-5p. The pmirGL3 reporter vectors were cloned with the wild-type hsa_circ_0086735 with the potential miR-1296-5p binding sites to construct WT-circ plasmids or mutant of these sites to construct MUT-circ plasmids, and then transfected into MCF-7 cells with miR-1296-5p mimic (mm-miR) or inhibitor (in-miR) or corresponding negative controls (mm-NC or in-NC). **(D)** *In vitro* pull-down of hsa_circ_0086735 with biotin-labeled miR-1296-5p (biotin-miR) or negative control (biotin-NC) in ZR-75-1 cells. **(E)** Predicted complementary binding sites of miR-1296-5p against STAT1. **(F)** Luciferase reporter assays confirm the binding between miR-1296-5p and STAT1. The pmirGL3 reporter vectors were cloned with the wild-type STAT1 with the potential miR-1296-5p binding sites to construct WT-STAT1 plasmids or mutant of these sites to construct MUT-STATA1 plasmids, and then transfected into ZR-75-1 cells with miR-1296-5p mimic (mm-miR) or inhibitor (in-miR) or corresponding negative controls (mm-NC or in-NC). **(G)** *In vitro* pull-down of STAT1 mRNA with biotin-labeled miR-1296-5p (biotin-miR) or negative control (biotin-NC) in MCF7 cells. **(H)** The expression level of miR-1296-5p was reversely related to that of STAT1. ***p < 0.001.

To confirm the interaction between hsa_circ_0086735 and miR-1296-5p, as well as miR-1296-5p and STAT1, Luciferase reporter assay and RNA pull-down assay, were introduced. The binding sites between hsa_circ_0086735 and miR-1296-5p were shown in Figure 7A, to gain the specific sequence of the combination of the two and thus to construct Luciferase report plasmids and sequence used in RNA pull-down assay. The expression of hsa_circ_0086735 was inversely correlated to that of miR-1296-5p (Figure 7B). Then the plasmids containing mutant- or wild-type hsa_circ_0086735 were used to study the influence of miR-1296-5p dysregulation on their luciferase activity. As expected, the reporter that carried binding sites was significantly influenced by miR-1296-5p mimics or inhibitors in MCF7 cells, whereas a luciferase reporter without binding was negligible (*p* < 0.001, Figure 7C). Then, biotin-labeled RNA pull-down assays demonstrated that biotin-labeled specific miR-1296-5p probes could pull down abundant hsa_circ_0086735 in ZR-75-1 cells (*p* < 0.001, Figure 7D). We simultaneously tested the binding of miR-1296-5p to STAT1 (Figure 7E). MiR-1296-5p was inversely expressed with STAT1 mRNA (Figure 7F). With Luciferase reporter assay, we assessed the binding of the wild-STAT1-containing reporter to miR-1296-5p and found that wild-type STAT1 resulted in repression of fluorescence intensity by miR-1296-5p mimics or induction by a miR-1296-5p inhibitor (*p* < 0.001, Figure 7G). RNA pull-down assay confirmed their binding (*p* < 0.001, Figure 7H).

### Hsa_circ_0086735-miR-1296-5p-STAT1 axis promoted luminal breast cancer progression

**FIGURE 8:**
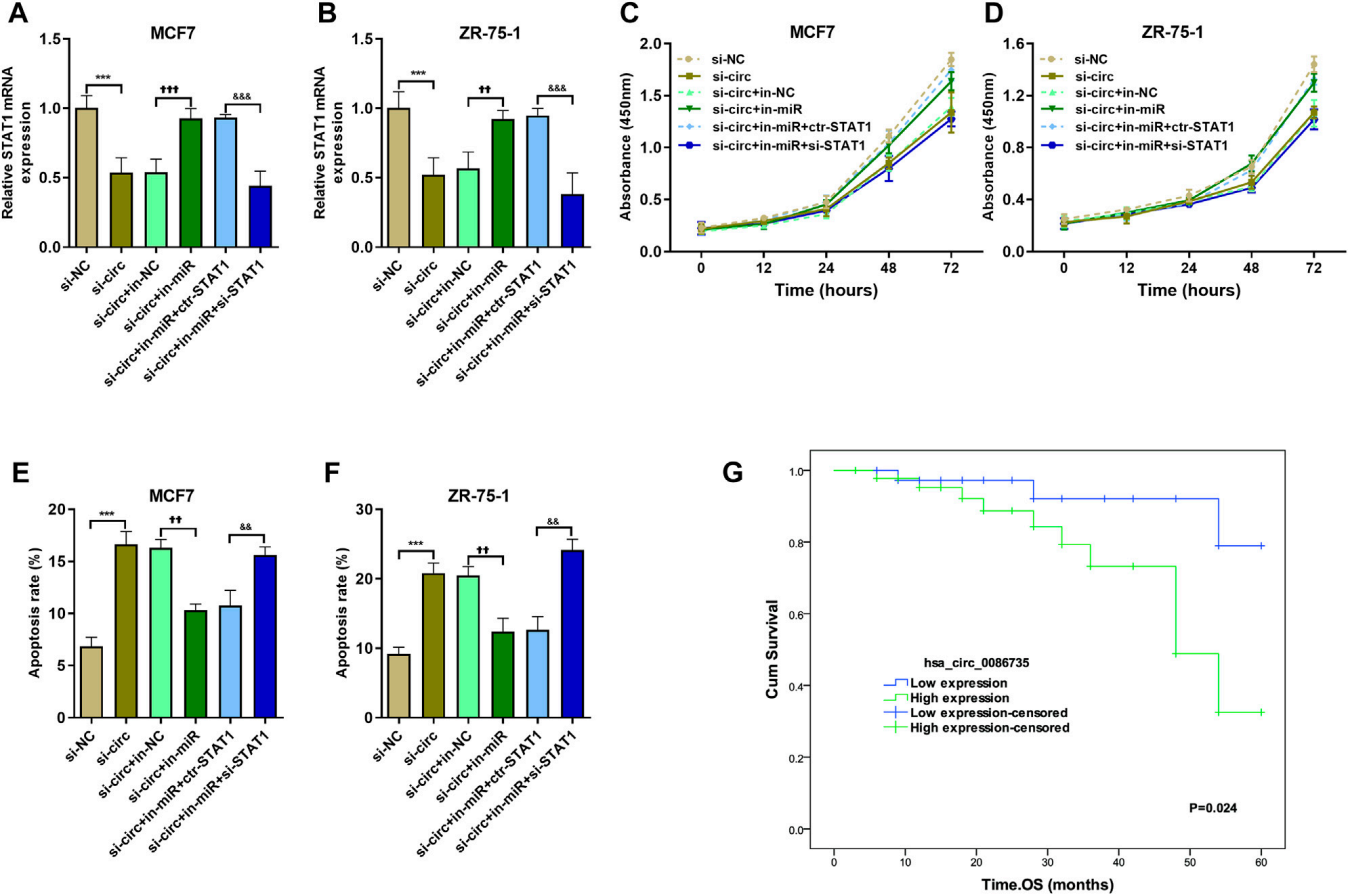
The effect of hsa_circ_0086735-miR-1296-5p-STAT1 axis on proliferation and apoptosis of luminal-subtype cell lines. **(A)** and **(B)** The transfection efficiency was confirmed by qRT-PCR analysis. CCK8 was performed on MCF7 **(C)** and ZR-75-1 **(D)** cells. Apoptosis assay by AnnexinV-FITC/PI staining for MCF7 **(E)** and ZR-75-1 **(F)** cells. **(G)** Overall survival of 87 breast cancer patients whose tumors had high or low hsa_circ_0086735 expression. ***p < 0.001, compared with si-NC group. ^††^ *p* < 0.01, ^†††^ *p* < 0.001, compared with si-circ+in-NC group. ^&&^ *p* < 0.01, ^&&&^ *p* < 0.001, compared with si-circ+in-miR + ctr-STAT1 group. si-NC: negative siRNA control for hsa_circ_0086735; si-circ: siRNA for hsa_circ_0086735; in-NC: inhibitor negative control; in-miR: miR-1296-5p inhibitor; ctr-STAT1: negative siRNA control for STAT1; si-STAT1: siRNA for STAT1.

To determine whether hsa_circ_0086735-miR-1296-5p-STAT1 axis was associated with luminal breast cancer cell function or patients’ survival, we assessed cell proliferation, cell apoptosis, and patients’ overall survival. Cell transfection was verified by the expression of STAT1 mRNA (Figures 8A,B). We observed a robust reduction in the proliferation of luminal breast cancer cells if hsa_circ_0086735 was individually inhibited, but increased again if both hsa_circ_0086735 and miR-1296-5p were inhibited until there was another reversal if hsa_circ_0086735, miR-1296-5p, and STAT1 were all inhibited (Figures 8C,D). The opposite trend occurs in cell apoptosis suggesting that hsa_circ_0086735 and STAT1 deficiency induced the apoptotic capacity, while miR-1296-5p inhibition reduced the apoptotic capacity (Figures 8E,F). The mean level of hsa_circ_0086735 (1.66) was defined as the cutoff for grouping of high expression and low expression. Next, high hsa_circ_0086735 was significantly correlated with patients’ histological type, tumor grade, molecular phenotype, and baseline Ki67 (Table 1). Additionally, high hsa_circ_0086735 was associated with poor overall survival in luminal breast cancer patients by Kaplan-Meier analysis (Figure 8G) and Multivariate Cox proportional hazards analysis (Table 2; HR 11.889, *p* = 0.007).

**TABLE 1:** Relations of hsa_circ_0086735 to main histopathological characteristics.

variables	Low hsa_circ_0086735 (n = 40)	High hsa_circ_0086735 (n = 47)	P-value
Categorical variables	Chi-square test		
Histological type			0.032
Ductal	35	32	
Lobular/Others	5	15	
Tumor Grade			
G1	22	12	0.005
G2/G3	18	35	
Molecular phenotype			
Luminal A	28	21	0.018
Non-Luminal A	12	26	
Continuous Variables	Spearman coefficient		
Age	0.114		0.294
Baseline Ki67 (log2)*	0.548		<0.001
Estrogen receptor*	0.046		0.672
Progesterone receptor*	0.045		0.679

*Percentage of tumor cells staining positively at immunohistochemistry.

**TABLE 2:** Multivariable analysis of predictors for the prognosis of overall survival and distant metastasis-free survival (DMFS).

Parameters	Overall survival			DMFS		
	HR	95% CI	*p*-value	HR	95% CI	*p*-value
hsa_circ_0086735	11.889	1.942–72.797	0.007	6.945	1.875–25.726	0.004
Histological type	3.679	0.959–14.120	0.058	2.196	0.749–6.437	0.152
Tumor Grade	9.757	1.021–93.226	0.048	3.653	0.932–14.313	0.063
Molecular phenotype	4.542	0.893–23.092	0.068	2.247	0.687–7.346	0.181
Age	2.505	0.650–9.653	0.182	1.742	0.612–4.955	0.298
Baseline Ki67 (log2)*	7.051	1.465–33.936	0.015	4.755	1.461–15.476	0.010
Estrogen receptor *	2.229	0.508–9.780	0.288	2.420	0.782–7.491	0.125
Progesterone receptor*	1.687	0.479–5.937	0.415	1.546	0.562–4.259	0.399

HR, hazard ratios; CI, confidence intervals.

### Hsa_circ_0086735-miR-1296-5p-STAT1 axis affects tamoxifen sensitivity

**FIGURE 9:**
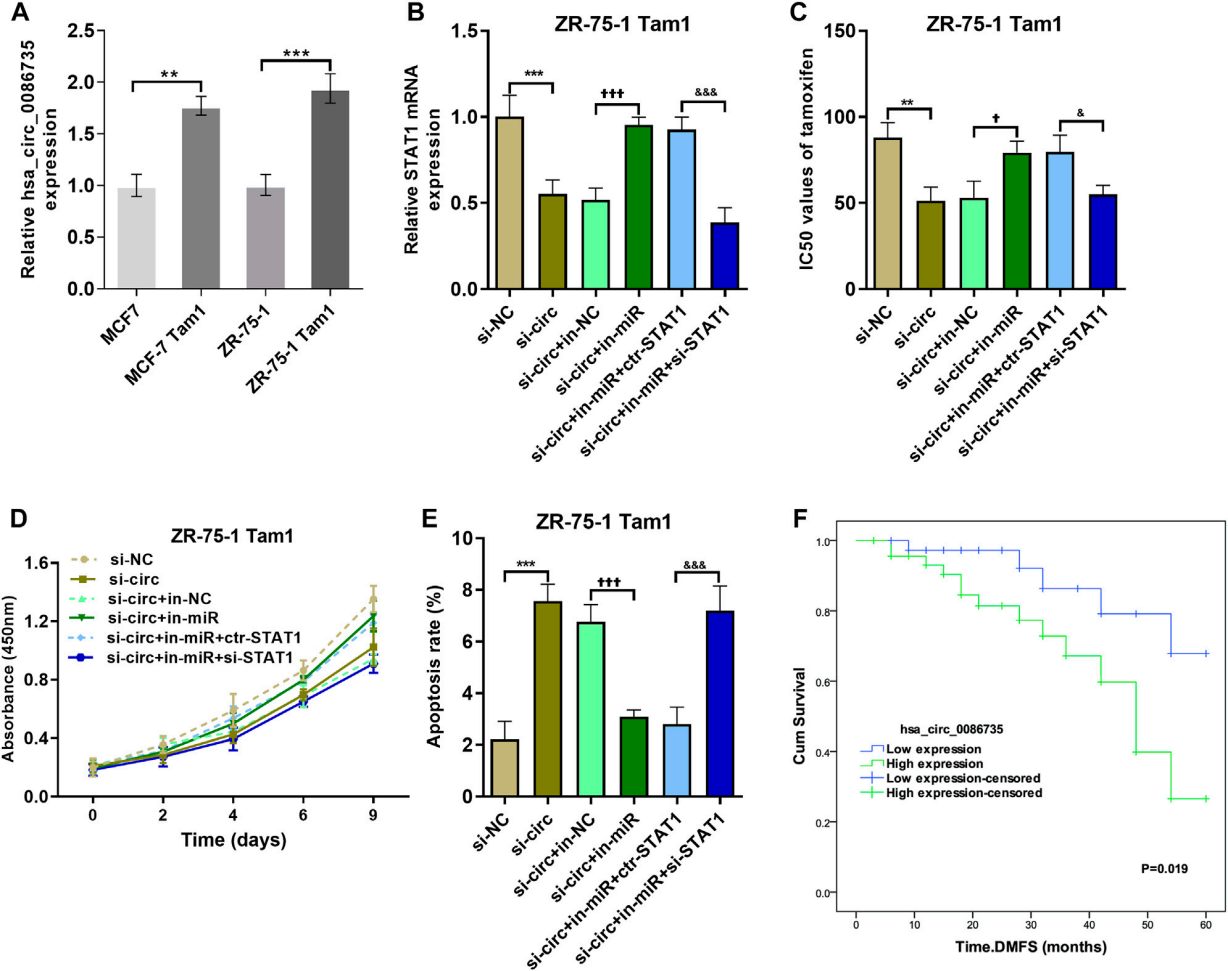
The effect of hsa_circ_0086735-miR-1296-5p-STAT1 axis on the proliferation of tamoxifen-resistant cells. **(A)** Hsa_circ_0086735 was upregulated in tamoxifen-resistant cells. **(B)** The transfection efficiency was confirmed by qRT-PCR analysis. **(C)** The IC50 values of different cell lines. **(D)** The cell proliferation by CCK-8 assay. **(E)** Apoptosis assay by AnnexinV-FITC/PI staining. **(F)** Distant metastasis-free survival of 87 breast cancer patients whose tumors had high or low hsa_circ_0086735 expression. ***p < 0.001, compared with si-NC group. ^††^ *p* < 0.01, ^†††^ *p* < 0.001, compared with si-circ+in-NC group. ^&&^ *p* < 0.01, ^&&&^ *p* < 0.001, compared with si-circ+in-miR + ctr-STAT1 group. si-NC: negative siRNA control for hsa_circ_0086735; si-circ: siRNA for hsa_circ_0086735; in-NC: inhibitor negative control; in-miR: miR-1296-5p inhibitor; ctr-STAT1: negative siRNA control for STAT1; si-STAT1: siRNA for STAT1.

To determine whether hsa_circ_0086735-miR-1296-5p-STAT1 axis contributes to tamoxifen resistance, the area under the ROC curve (AUC) for STAT1, along with the other three genes in circRNA-miRNA-mRNA-pathway network, discriminating the non-response and response groups, was obtained from CTR-DB datasets (Supplementary Figure S1). Then, the expression of hsa_circ_0086735 was determined as an upregulated status in tamoxifen-resistant cell lines compared to that in the parental cell lines (Figure 9A). ZR-75-1 Tam1 cell line with a higher hsa_circ_0086735 expression was transfected for the subsequent experiments (Figure 9B). ZR-75-1 Tam1 developed a vulnerability to tamoxifen after hsa_circ_0086735 knockdown or hsa_circ_0086735/miR-1296-5p/STAT1 inhibition but become resistant to tamoxifen when hsa_circ_0086735 and miR-1296-5p were simultaneously inhibited, presented as the change in IC50 values (Figure 9C). Subsequent functional assays suggested that inhibited hsa_circ_0086735 reduced the cell proliferation potential, while miR-1296-5p can weaken this effect but STAT1 can consolidate it (Figure 9D). The induction of hsa_circ_0086735 on cell apoptosis rate can be abolished by miR-1296-5p and reproduced with the inhibition of STAT1 (Figure 9E). In patients treated with tamoxifen, high hsa_circ_0086735 was associated with poor distant metastasis-free survival (DMFS) (Figure 9F; Table 2, HR 6.945, *p* = 0.004).

## Discussion

The discovery of circRNA subsets has added a new layer of complexity to the alterations of gene expression associated with epigenetic changes, primarily via the ceRNA network comprised of various circRNA-miRNA-mRNA axes (Shen et al., 2015; Fontemaggi et al., 2021). The dysfunction of circRNA-miRNA-mRNA regulatory signal represents another important epigenetic-controlled mechanism of the pathogenic gene expression in human cancer, which are important nodes in cancer development (Tang et al., 2021; Mo et al., 2022). Mapping the circRNA-miRNA-mRNA network is an important way to elucidate the regulatory role of circRNA being investigated (Rengganaten et al., 2020; Pang et al., 2022). In this work, we screened luminal-specific and tamoxifen-resistant-related circRNAs, as well as differentially expressed miRNAs and mRNAs, from GEO breast cancer datasets. The differentially expressed circRNAs, miRNAs, and mRNA were subsequently used in the mapping of a novel circRNA-miRNA-mRNA network. Finally, the obtained hsa_circ_0086735-miR-1296-5p-STAT1 was proved to promote the progression of luminal breast cancer and tamoxifen resistance.

Growing evidence has validated the ceRNA function of circRNAs in cancers (Becatti et al., 2023). For instance, circularLRRC7 can exert as a potential glioblastoma suppressor by functioning as ceRNA for miR-1281 (Kong et al., 2021). In the current study, microarray datasets from the GEO database were downloaded and analyzed to identify robust differentially expressed circRNAs, miRNAs, and mRNAs that are specific to luminal-subtype breast cancer. Using the GEO2R method and other online analysis plates, our study systematically integrated multiple microarray datasets on breast cancer in GEO. The PPI network was constructed and used to screen hub genes. GO and KEGG enrichment analyses were conducted to annotate the functions of differentially expressed genes. The network was constructed, and the pathway network composed of hub genes was mapped. Based on the circRNA-miRNA-mRNA-pathway network, hsa_circ_0086735-miR-1296-5p-STAT1 axis was used for verification. The expression levels of hsa_circ_0086735, miR-1296-5p, and STAT1 mRNA were confirmed by qRT-PCR in luminal-subtype tissues and cell lines. The interactions among them were verified by Luciferase reporter assay and RNA pull-down assay.

A proven research result is that circRNA can influence cancer cell function. For example, the upregulation of circ_0008812 and circ_0001583 can contribute to the proliferation of breast cancer cells (Lin et al., 2022). Though hsa_circ_0086735 has few reports, hsa_circUBAP2 has been reported as an upregulated circRNA in breast cancer (Ma et al., 2021), just like the upregulated expression in luminal-subtype in this study. We also revealed the promoting role of hsa_circ_0086735 in breast cancer cell growth, which was in line with its role in glioma (Wang et al., 2021). The prognosis significance of hsa_circ_0086735 was verified in our 87 patients, which was mentioned in a previous meta-analysis (Ma et al., 2021). MiR-1296-5p has been reported to decrease ERBB2-positive breast cancer and exerts a tumor-suppressive function in breast cancer (Chen et al., 2017). The other known circRNAs that can target miR-1296-5p include hsa_circs_0000517, hsa_circ_0017639, and circ_0048764 (Zang et al., 2020; Chang et al., 2022). STAT1 was verified to accelerate breast cancer via deregulating homeostasis of the tumor microenvironment (Zellmer et al., 2017; Hou et al., 2018). Many cancers, including cancer, demonstrate. Inhibiting STAT1 activation, along with facilitating STAT3 activation, can aid the removal of breast malignant cells via EGFR moderation (Concha-Benavente et al., 2013). Therefore, the hsa_circ_0086735-miR-1296-5p-STAT1 axis can promote the cell proliferation and inhibited cell apoptosis of luminal-subtype breast cancer (Supplementary Figure S2).

CircRNAs can influence breast cancer cell response to endocrine therapies by mechanisms involving different estrogen signaling via modulating the expression of proteins acting as coregulators of estrogen signaling (Molibeli et al., 2021; Bahramy et al., 2023). Possible involvements of hsa_circ_0086735-miR-1296-5p-STAT1 axis in resistance of tamoxifen were investigated in this work. We revealed that hsa_circ_0086735 contributed to the resistance of tamoxifen. miR-1296-5p inhibitor has been discovered to restore the decreased cisplatin resistance in cisplatin-resistant non-small cell lung cancer cells (Chang et al., 2022). In this study, miR-1296-5p inhibitor restored the tamoxifen resistance in ZR-75-1 Tam1 cells. Depletion of STAT1 can decrease levels of ERα protein and cell proliferation in tamoxifen-resistant cell line LCC2 (Hou et al., 2018). A previous study has identified that suggest that increased STAT1 signaling is important in endocrine resistance and that STAT inhibitors may represent potential therapies in endocrine-resistant breast cancer (Huang et al., 2014; Chen et al., 2023). STAT1/c-Myc pathway is involved in POR-induced TAM-resistant breast cancer (Chen et al., 2023). The present study reveals a possible tamoxifen-resistant mechanism by which hsa_circ_0086735 may sponge miR-1296-5p, indirectly moderating STAT1 and contributing to tamoxifen resistance. Targeting of hsa_circ_0086735 may be a potential treatment strategy for tamoxifen-resistant luminal breast cancers.

A circRNA-miRNA-mRNA network was mapped in luminal breast cancer in this work. Hsa_circ_0086735-miR-1296-5p-STAT1 axis in the network can promote cancer progression and resistance to tamoxifen. Hsa_circ_0086735-miR-1296-5p-STAT1 axis may serve as therapeutic considerations to eradicate tamoxifen -resistance in breast cancer.

## Data Availability

The original contributions presented in the study are included in the article/Supplementary Material, further inquiries can be directed to the corresponding author.
