# Supplementary figures and images for: Identification of circRNA-miRNA-mRNA network in luminal breast cancers by integrated analysis of microarray datasets
Source: Front Mol Biosci. 2023 Apr 28;10:1162259. doi: 10.3389/fmolb.2023.1162259 (PMC10175596; doi:10.3389/fmolb.2023.1162259)

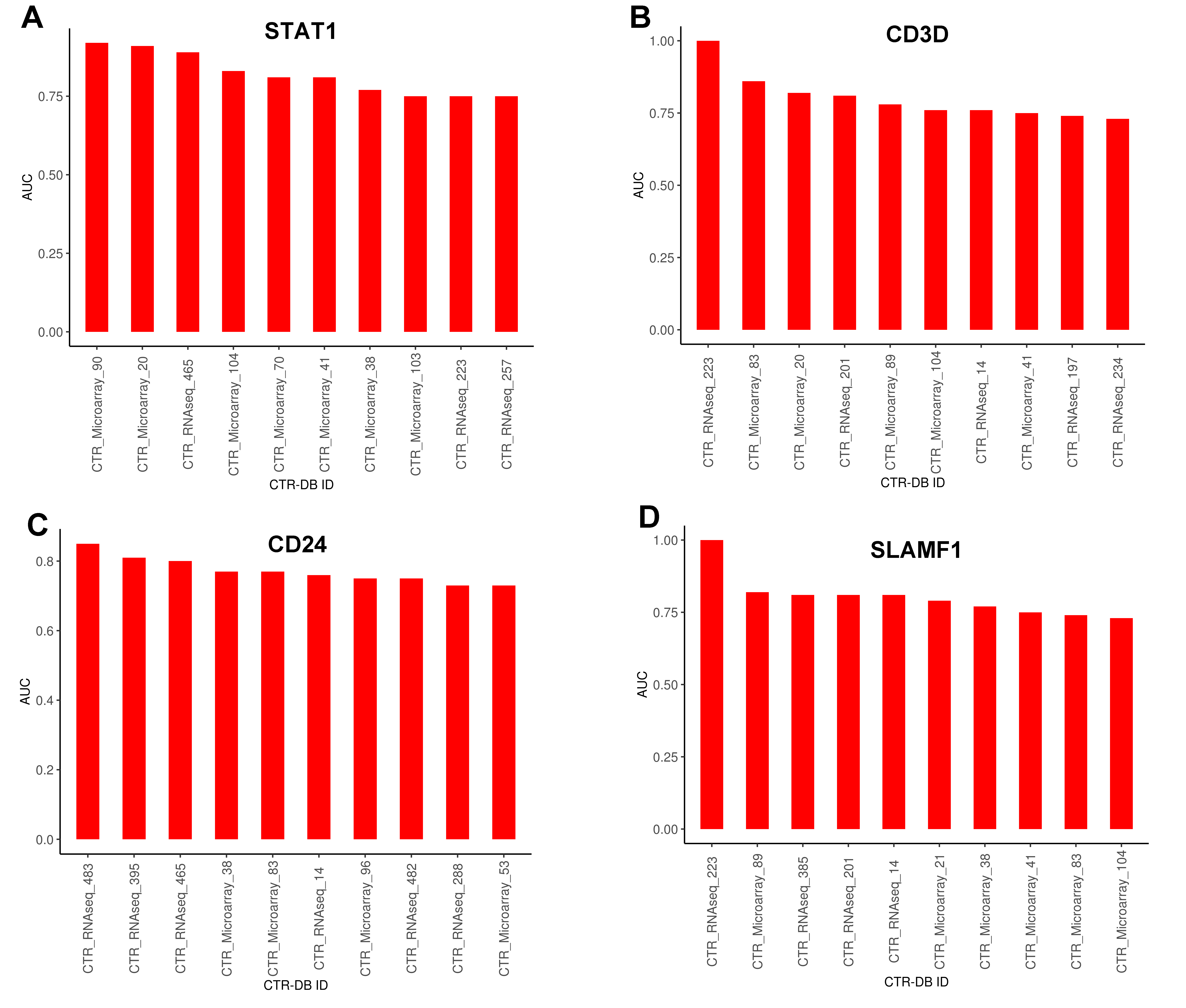

Supplement: Supplementary file 1 [file Image1.TIFF]

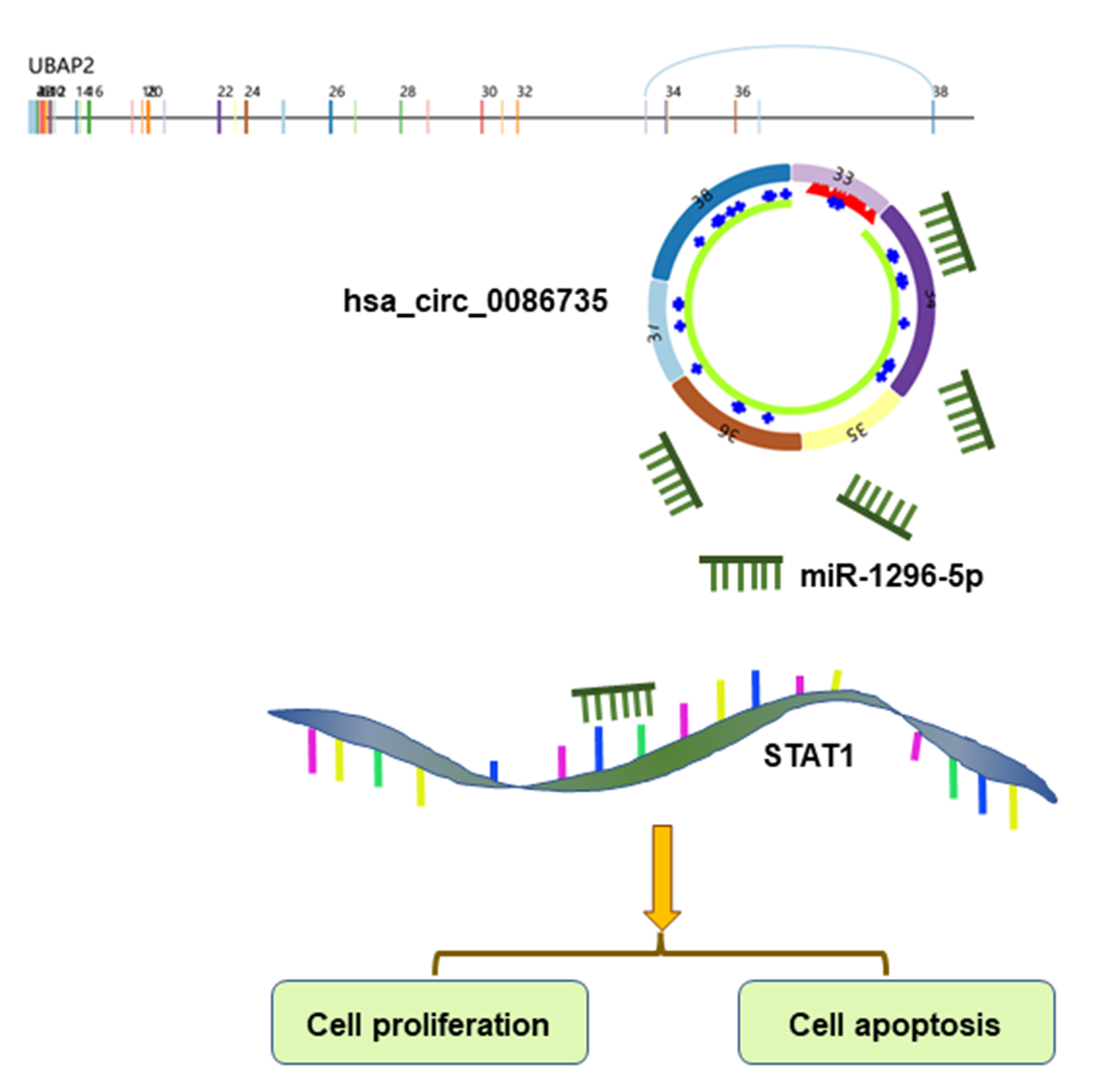

Supplement: Supplementary file 3 [file Image2.TIF]
