# Supplementary material for: Identification of circRNA-miRNA-mRNA network in luminal breast cancers by integrated analysis of microarray datasets
Source: Front Mol Biosci. 2023 Apr 28;10:1162259. doi: 10.3389/fmolb.2023.1162259 (PMC10175596; doi:10.3389/fmolb.2023.1162259)
Supplement: Supplementary file 2 [file Table1.DOCX]

**Table 1** Relations of hsa_circ_0086735 to main histopathological characteristics

| variables | Low hsa_circ_0086735  (n = 40) | High hsa_circ_0086735  (n = 47) | *P-value* |
| --- | --- | --- | --- |
| Categorical variables | Chi-square test |  |  |
| Histological type |  |  | 0.032 |
| Ductal | 35 | 32 |  |
| Lobular/Others | 5 | 15 |  |
| Tumor Grade |  |  |  |
| G1 | 22 | 12 | 0.005 |
| G2/G3 | 18 | 35 |  |
| Molecular phenotype |  |  |  |
| Luminal A | 28 | 21 | 0.018 |
| Non-Luminal A | 12 | 26 |  |
| Continuous Variables | Spearman coefficient |  |  |
| Age | 0.114 |  | 0.294 |
| Baseline Ki67 (log2) * | 0.548 |  | <0.001 |
| Estrogen receptor * | 0.046 |  | 0.672 |
| Progesterone receptor * | 0.045 |  | 0.679 |

*Percentage of tumor cells staining positively at immunohistochemistry.
