# Supplementary material for: Identification of circRNA-miRNA-mRNA network in luminal breast cancers by integrated analysis of microarray datasets
Source: Front Mol Biosci. 2023 Apr 28;10:1162259. doi: 10.3389/fmolb.2023.1162259 (PMC10175596; doi:10.3389/fmolb.2023.1162259)
Supplement: Supplementary file 4 [file Table2.DOCX]

**Table 2** Multivariable analysis of predictors for the prognosis of overall survival and distant metastasis free survival (DMFS).

| Parameters | overall survival | | |  | DMFS | | |
| --- | --- | --- | --- | --- | --- | --- | --- |
|  | HR | 95%CI | *P*-value |  | HR | 95%CI | *P*-value |
| hsa_circ_0086735 | 11.889 | 1.942-72.797 | 0.007 |  | 6.945 | 1.875-25.726 | 0.004 |
| Histological type | 3.679 | 0.959-14.120 | 0.058 |  | 2.196 | 0.749-6.437 | 0.152 |
| Tumor Grade | 9.757 | 1.021-93.226 | 0.048 |  | 3.653 | 0.932-14.313 | 0.063 |
| Molecular phenotype | 4.542 | 0.893-23.092 | 0.068 |  | 2.247 | 0.687-7.346 | 0.181 |
| Age | 2.505 | 0.650-9.653 | 0.182 |  | 1.742 | 0.612-4.955 | 0.298 |
| Baseline Ki67 (log2) * | 7.051 | 1.465-33.936 | 0.015 |  | 4.755 | 1.461-15.476 | 0.010 |
| Estrogen receptor * | 2.229 | 0.508-9.780 | 0.288 |  | 2.420 | 0.782-7.491 | 0.125 |
| Progesterone receptor * | 1.687 | 0.479-5.937 | 0.415 |  | 1.546 | 0.562-4.259 | 0.399 |

HR, hazard ratios. CI, confidence intervals.
