# Supplementary material for: Identification of circRNA-miRNA-mRNA network in luminal breast cancers by integrated analysis of microarray datasets
Source: Front Mol Biosci. 2023 Apr 28;10:1162259. doi: 10.3389/fmolb.2023.1162259 (PMC10175596; doi:10.3389/fmolb.2023.1162259)
Supplement: Supplementary file 5 [file Table3.DOCX]

**Supplementary Table 1 Top 10 in protein-protein interaction network.**

| Rank | Name | Score |
| --- | --- | --- |
| 1 | PTPRC | 17 |
| 2 | STAT1 | 10 |
| 3 | IL7R | 10 |
| 4 | LCK | 9 |
| 5 | SLAMF1 | 7 |
| 6 | CCR5 | 7 |
| 7 | ITK | 6 |
| 8 | CD38 | 6 |
| 9 | CD3D | 5 |
| 10 | CD24 | 5 |
